# Case report: NAFLD and maple syrup urine disease: Is there an interplay between branched-chain amino acids and fructose consumption?

**DOI:** 10.3389/fped.2022.933081

**Published:** 2022-10-10

**Authors:** Helena Moreira-Silva, Sandra Ferreira, Manuela Almeida, Isabel Gonçalves, Maria Augusta Cipriano, J. R. Vizcaíno, Ermelinda Santos-Silva, Esmeralda Gomes-Martins

**Affiliations:** ^1^Pediatric Gastroenterology Unit, Centro Materno Infantil do Norte, Centro Hospitalar Universitário do Porto, Porto, Portugal; ^2^Hepatology and Pediatric Liver Transplantation Unit, Hospital Pediátrico de Coimbra, Centro Hospitalar e Universitário de Coimbra, Coimbra, Portugal; ^3^Pediatric Metabolic Diseases Unit, Centro de Referência de Doenças Hereditárias do Metabolismo, Centro Hospitalar Universitário do Porto, Porto, Portugal; ^4^Pathology Department, Centro Hospitalar e Universitário de Coimbra, Coimbra, Portugal; ^5^Anatomic Pathology Service, Pathology Department, Centro Hospitalar Universitário do Porto, Porto, Portugal

**Keywords:** NAFLD (non-alcoholic fatty liver disease), graft liver steatosis, fructose, recurrent NAFLD, maple syrup urine disease (MSUD), liver transplant

## Abstract

**Background:**

The worldwide increase in pediatric overweight and obesity, in parallel with the global increase in the consumption of sucrose and fructose, is associated with non-alcoholic fatty liver disease (NAFLD). Elevated branched-chain amino acids (BCAAs) are a metabolic feature related to obesity and an early risk factor for insulin resistance and NAFLD. However, few studies have assessed metabolic risk factors and nutritional status in maple syrup urine disease (MSUD) patients under restricted BCAA and high carbohydrate diets.

**Methods and results:**

Herein, we present a pilot report of a 17-year-old boy with classic MSUD with poor diet compliance and high fructose consumption, mainly during early adolescence. At that time, he was overweight and developed features of metabolic syndrome, including persistently elevated liver enzymes and hepatic steatosis. He underwent liver transplantation at the age of 13 years to prevent the risk of progressive cognitive impairment. Two months later, NAFLD relapsed in the graft, despite a better BCAA balance and weight loss. Nevertheless, 6 months after dietary restriction of fructose consumption, NAFLD had sustainably improved.

**Conclusion:**

Childhood overweight and fructose overconsumption are wellestablished driving forces in the development of pediatric NAFLD. However, their role in the early onset and progression of NAFLD in the allograft remains to be established. Furthermore, it is not known whether the dysmetabolic state associated with elevated BCAAs may be contributory. Further studies are required with a cohort of MSUD subjects to validate our findings and to ascertain the possible interaction between a BCAA imbalance and dietary intake in the development of NAFLD.

## Introduction

Maple syrup urine disease (MSUD, OMIM 248600) is an autosomal recessive inherited metabolic disorder (IMD) caused by biallelic variants in one of the three genes: *BCKDHA, BCKDHB*, and *DBT*. The resultant branched-chain α-ketoacid dehydrogenase (BCKDH) deficiency impairs the metabolism of the branched-chain amino acids (BCAAs), leucine, isoleucine, and valine (1, 2). Lifetime stringent dietary therapy (protein-restricted and synthetic amino acid supplements) is the cornerstone of management, but is challenging and does not fully prevent metabolic crisis and cognitive and psychiatric disabilities (1, 3). Orthotopic liver transplantation (OLT) restores about 10% of BCKDH activity, allowing better biochemical control under a partially unrestricted diet and providing protection from metabolic crises (1, 4).

Despite the evidence suggesting that elevated BCAAs are a metabolic feature associated with obesity and an early risk factor for insulin resistance and non-alcoholic fatty liver disease (NAFLD) (5, 6), few studies have assessed the nutritional status of patients with IMDs. However, it is accepted that being overweight may be a concern and that the reduction of protein intake can predispose to the consumption of a high-fat, high-sugar (from sucrose and/or high fructose) diet (7). Additionally, the worldwide increase in pediatric overweight and obesity parallels the global diet modifications including the rise of sucrose (table sugar) and fructose consumption, mainly present in sugar-sweetened beverages (8–10). The hepatic oversupply of these substrates is a major mediator of non-alcoholic fatty liver disease (or “*fructoholic* liver disease”) (8, 9, 11–13), correlating with the severity of hepatic fibrosis in a dose-dependent manner (14, 15).

Herein, we present a pilot case report of a child with classic MSUD with poor metabolic control who developed NAFLD and other features of metabolic syndrome. He underwent OLT to prevent further cognitive decline. After OLT, besides achieving a better BCAA balance, he developed recurrent NAFLD, which only regressed after the restriction of fructose consumption. We highlight the role of fructose on NAFLD pathogenesis and related metabolic syndrome and speculate on the possible interaction between BCAA and nutritional status (beyond BCAA-restricted diets).

## Case description

Our patient was a 17-year-old male, the second child of a non-consanguineous couple. Prenatal screening and delivery were unremarkable. On the 7th day of life, he was admitted due to poor feeding, hypotonia, and episodes of apnea. On admission, he presented with hypoglycemia (30 mg/dL), metabolic acidosis (Ph = 7.30), and increased urinary ketone bodies (3 + range on the dipstick test). The plasma amino acids chromatography revealed a marked elevation in BCAAs (leucine 1,889 μmol/L (normal range 47–109 μmol/L), isoleucine 432 μmol/L (normal range 27–94μmol/L), valine 895 μmol/L (normal range 8–246 μmol/L), and allo-isoleucine 257 μmol/L (normal range 1.2–3.4 μmol/L). A molecular study showed homozygous deletion of exons 2, 3, and 4 in the BCKDHA gene, establishing the diagnosis of classic MSUD.

Ventilatory support, intravenous high glucose, insulin infusion, and supplementation with oral BCAA-free formula were started, with normalization of leucine levels achieved on the 6th day of treatment. Neurological stability and normal oral intake enabled him to be discharged during the 3rd week of life.

The patient had regular a follow-up in our Metabolic Diseases Unit with nutritional, metabolic, and neurocognitive assessments. He was prescribed a long-term treatment consisting of guided dietary intake, with a strict low-protein diet and a BCAA-free formula to provide adequate macronutrients, prevent catabolism, and maintain plasma BCAA within the target treatment range. Basal leucine requirements were achieved using infant formula; valine and isoleucine supplements were added to maintain metabolic homeostasis.

Up to preschool age, he had good metabolic control and neurocognitive development was age-appropriate. In the following years, dietary treatment was not strictly followed along with overconsumption of sweetened beverages (juices and soft drinks) and a high content of simple carbohydrates/added sugar (processed foods). At the age of 7 years, he was overweight (BMI = 27 kg/m^2^, percentile >99th) and his blood chemistry was remarkable for hyperuricemia and intermittent elevated liver enzymes (1–2x upper limit of normal—ULN). During early adolescence, despite the absence of serious metabolic decompensations, chronic non-compliance with the BCAA-restricted diet led to persistently elevated leucine levels (400–500 μmol/L) and cognitive impairment (intelligence coefficient score below average). In parallel with obesity (abdominal predominant), other features of metabolic syndrome ensued hypertriglyceridemia (196 mg/dL), low HDL (35 mg/dL), high blood pressure, and peripheral insulin resistance.

At the age of 11 years, he was evaluated in our Pediatric Gastroenterology Unit due to persistently elevated liver enzymes (on admission: AST 93 UI/L, ALT 194 UI/L, GGT 208 UI/L). Abdominal ultrasound showed liver enlargement and a heterogeneous echostructure, compatible with diffuse hepatic steatosis. Further laboratory work-up excluded primary liver disease (autoimmune liver disease, hepatitis B and C, and alpha-1 antitrypsin deficiency).

**Figure 1:**
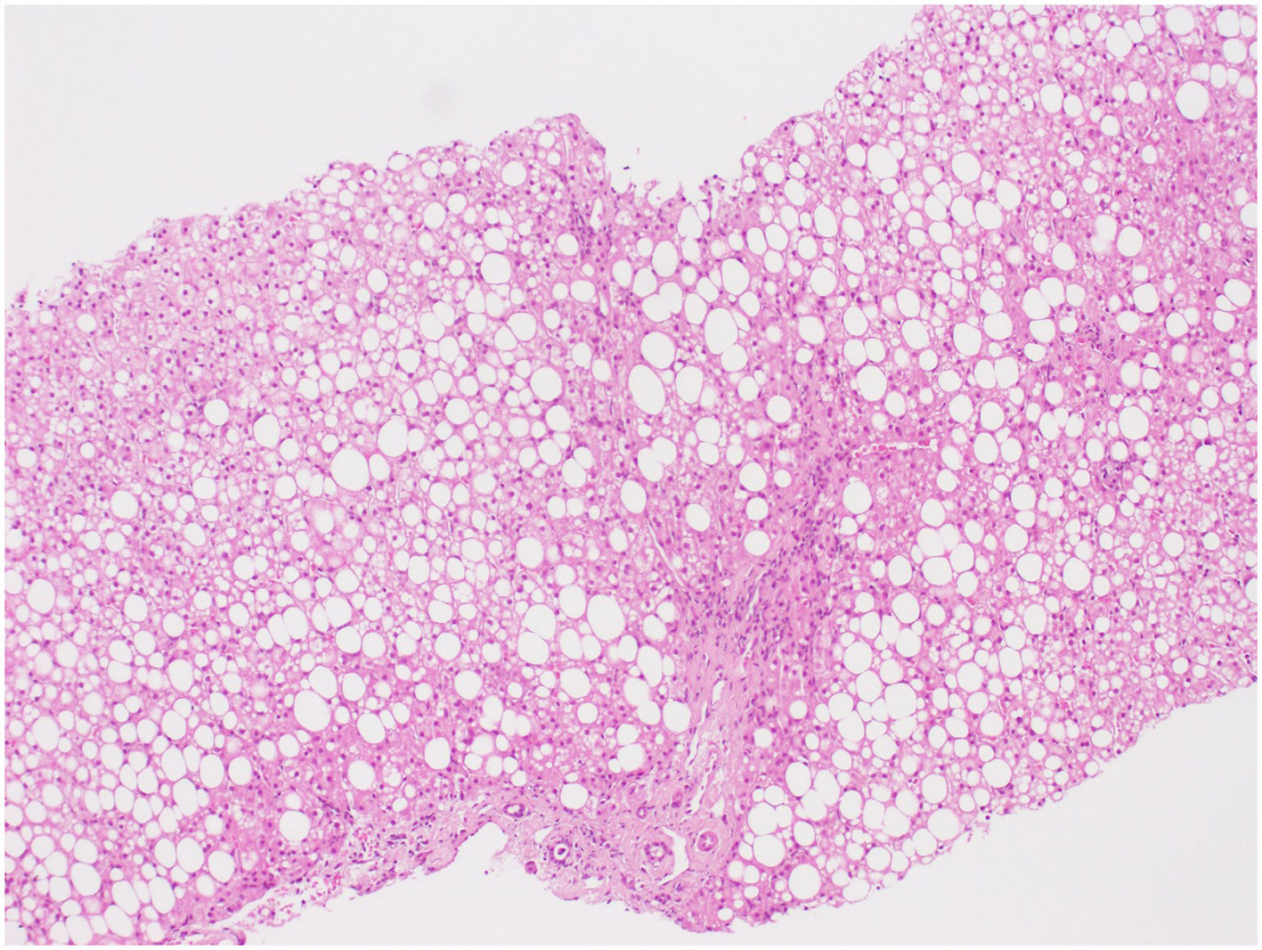
Hematoxylin and eosin (H&E) × 100. Photomicrograph illustrating predominantly macro-vesicular steatosis without hepatocyte ballooning or Mallory bodies; the presence of mononuclear inflammation and septal fibrosis.

**Figure 2:**
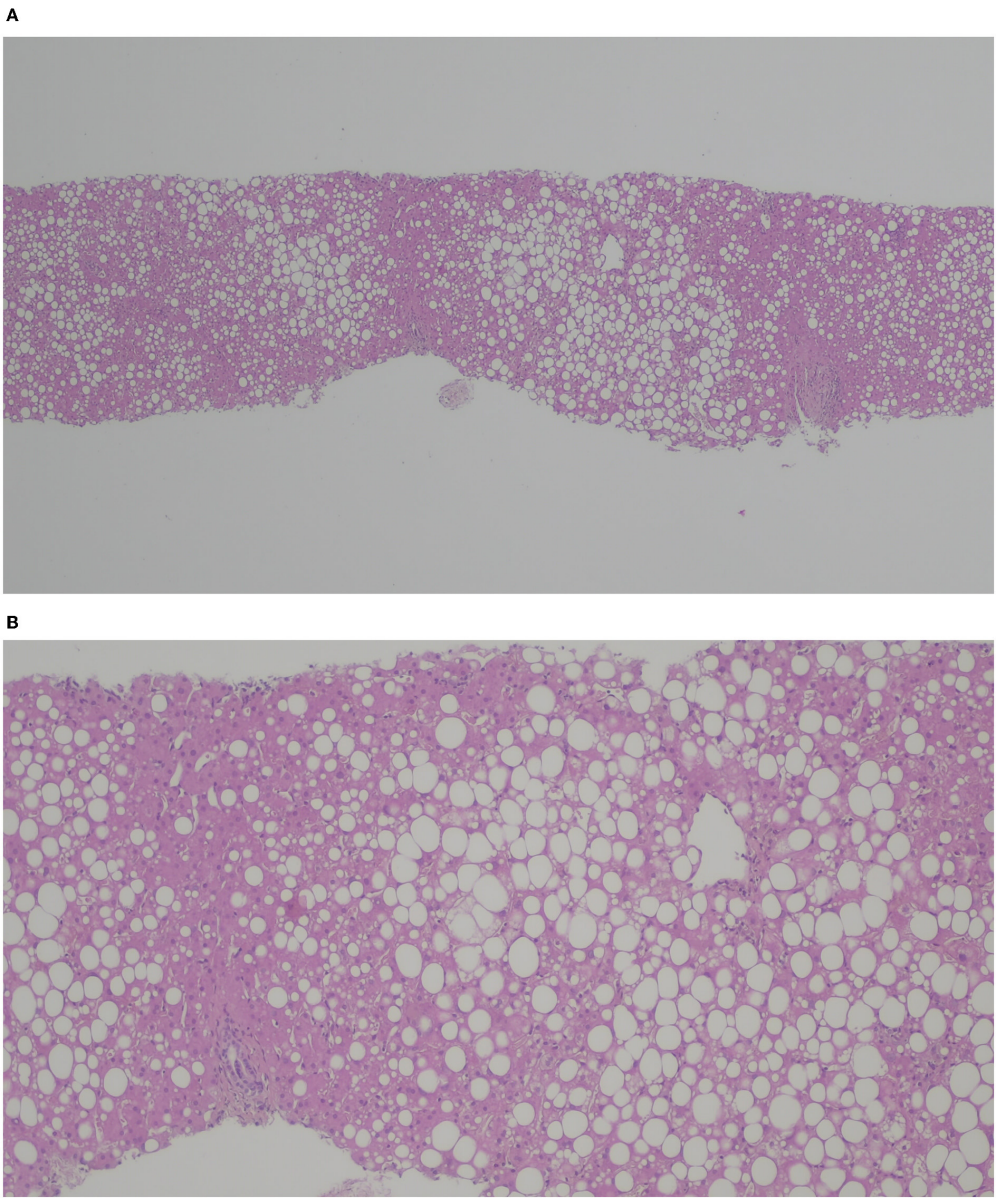
H&E **(A)** × 40, **(B)** x 100. Liver graft with macro- and meso-steatosis (40%); no features of graft rejection.

A serum level of ceruloplasmin of 18.2, and 20 mg/dL on repeat testing, triggered further work-up for Wilson's disease: 24-h urinary copper excretion was 0.798 μmol/day, and Kayser–Fleischer rings were absent, but hepatic copper concentration was 17.67 μmol/g of tissue. Genetic testing revealed simple heterozygosity in the ATP7B gene-c.1607T >C (p.Val536Ala)—Leipzig score of 4. Liver histopathology showed macro-vesicular steatosis (> 60%) and portal fibrosis (Figure 1). Poor metabolic control with progressive cognitive impairment culminated in OLT at the age of 13 (cadaveric ABO-matched young donor with no underlying medical disease; graft without steatosis). The postoperative period was uneventful, and he was discharged on the 13th day. The patient was under combined immunosuppression with tacrolimus (target blood trough levels of 6–10 ng/mL) and prednisolone for the first few months. Prednisolone was progressively tapered and stopped. During the following 3 months, he had a noticeable decrease in BCAA levels (leucine from 500 to 150 μmol/L), despite the increase in dietary proteins (natural protein intake >1 g/kg/day), and weight reduction. Regardless of this improvement, he maintained elevated transaminases (ALT 4xULN, AST, and GGT 2xULN), hyperuricemia (although lower than pretransplant levels), and hypertriglyceridemia. Liver graft histology revealed macro-steatosis relapse with ~40% hepatocytes affected, with no features of graft rejection (Figure 2). Sustained high sucrose and fructose intake, particularly sugar-sweetened beverages (> 100 g/day), along with a lack of physical exercise was identified as contributory factors.

**Figure 3:**
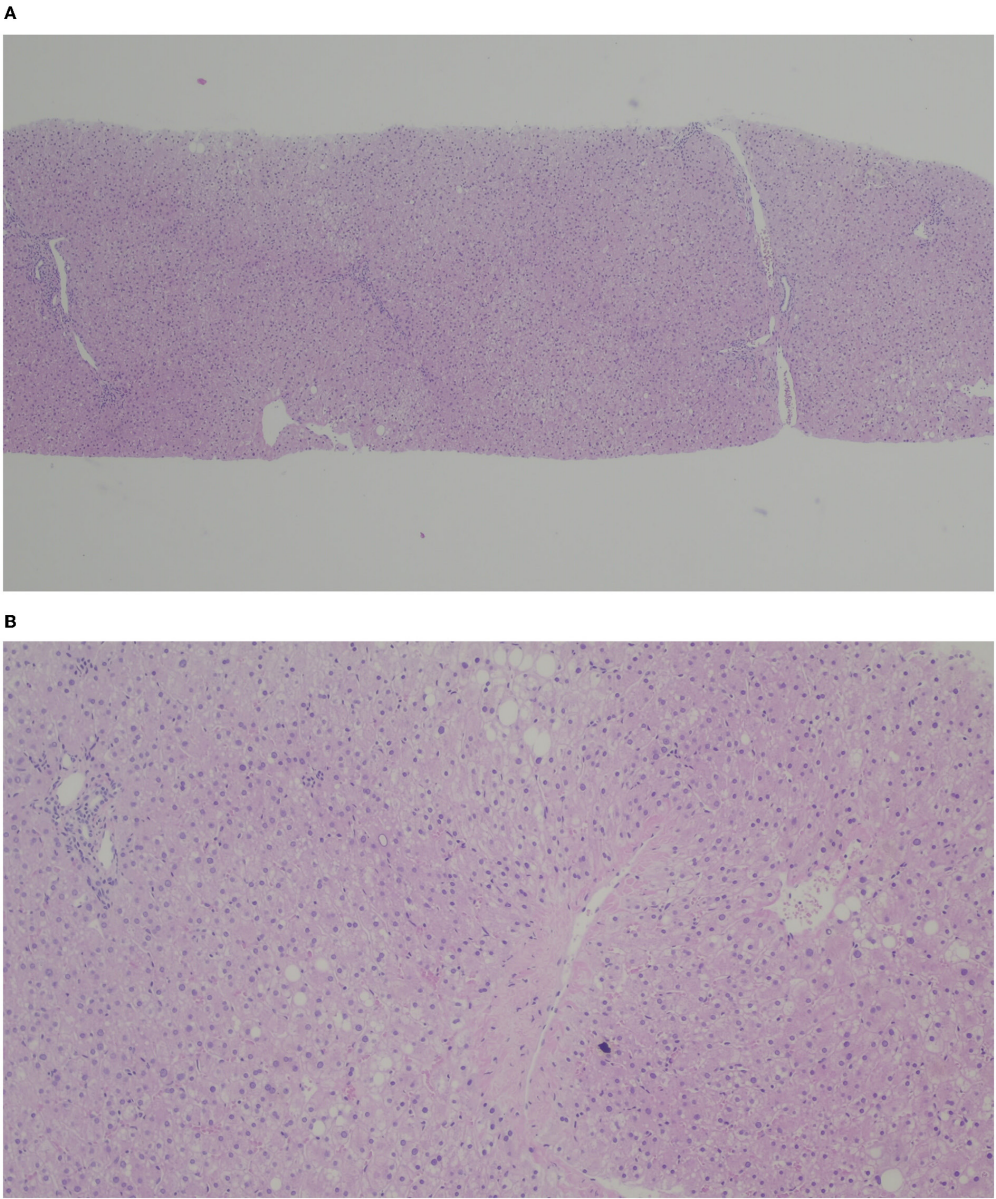
H&E **(A)** × 40, **(B)** x 100. Liver graft with micro and macrovacuolar steatosis (< 5%) with slight portal and centrolobular fibrosis.

Seven months post-OLT, after nutritional optimization with fructose reduction and an increase in daily physical activity, the patient had a BMI in the 85–97th percentile, normal transaminases, normal triglycerides, and leucine serum levels around 200 μmol/L. Although graft histology had improved, it still revealed 15–30% of macro-steatosis. Two years later, after sustained nutritional improvement along with weight reduction, he had normal liver enzymes and a graft histology with 5% of steatosis (Figure 3).

## Discussion

MSUD is a complex metabolic disorder due to a pathogenic defect in any BCKAD subunit, resulting in elevated BCAAs, leucine, isoleucine, and valine, and their corresponding α-ketoacids (2). In the classical form (< 3% residual enzyme activity), MSUD can present with metabolic encephalopathy manifested by acute ketoacidosis and neurological symptoms such as seizures, apnea, and coma within the first days of life (1, 3, 16). In Portugal, MSUD has been included in the newborn screening (NBS) program since 2006, thus allowing the identification of newborns either asymptomatically or in the early onset of their symptoms. Our patient, however, presented before the implementation of the NBS program, and thus, the diagnosis was based on the typical clinical presentation and biochemical data, confirmed by the molecular study.

Despite advances in early diagnosis with the NBS, patients with classic MSUD still remain at risk for chronic metabolic instability and cognitive impairment. BCAAs are important precursors for various physiologic processes, such as protein synthesis, gluconeogenesis, fatty acid synthesis, cholesterol synthesis, and cellular signaling (17). Leucine and its corresponding ketoacid appear to be the most neurotoxic (16), causing cerebral edema and lesions of hypomyelination in newborns and infants (18). In the older child, chronic amino acid imbalances pose a risk for abnormal brain imaging, intellectual disability, and hyperactivity/attention deficit (1, 19).

Current management of MSUD consists of dietary therapy and liver transplantation, which partially restore functional BCKDH activity (3). Although strict dietary treatment from infancy typically yields good outcomes, it requires the restriction of BCAAs by limiting natural protein in the diet and supplementation of essential amino acids and a low-protein diet. This restrictive diet is challenging to maintain, and management of each metabolic decompensation requires a high caloric intake (up to 150% of usual energy intake) yielding the patients at risk for nutritional imbalances (1, 20). Notably, dietary protein restriction (below 9% of total energy or <1.0 g/kg/day) and high carbohydrate intake (especially refined sugars) have been linked to excessive hepatic lipid accumulation and the development of NAFLD (21, 22).

The liver plays an essential role in maintaining nutritional homeostasis, and therefore, inadequate dietary intake can adversely affect body composition and biological functions. In this respect, in our patient, dietary compliance allowed favorable metabolic control, while ensuring adequate growth and a positive neurocognitive outcome until preschool years. Subsequent irregular compliance with the BCAA-restricted diet and poor metabolic control was probably responsible for his cognitive delay and a cofactor in the development of NAFLD and the associated metabolic dysfunction. Furthermore, he also maintained a calorically unrestricted diet containing beverages and processed foods that placed him at risk for metabolic and nutritional imbalance. In fact, this pattern of consumption is positively correlated with the development of obesity, NAFLD, and other features of metabolic syndrome in early adolescence: hypertriglyceridemia, high blood pressure, and insulin resistance (23).

NAFLD is becoming one of the most common causes of chronic liver disease in children in developed countries, and non-alcoholic steatohepatitis (NASH)-related cirrhosis is an increasingly recognized indication for OLT in adults (24). NAFLD, which is associated with insulin resistance, obesity, and hyperlipidemia, is considered the hepatic manifestation of metabolic syndrome. Recently, a proposal has been made to rename NAFLD as metabolic-associated fatty liver disease (MAFLD), due to the heterogeneous etiology and the aforementioned metabolic risks (25). Diet is an important and wellestablished modifiable risk factor for NAFLD. In fact, the epidemic of pediatric NAFLD parallels the increase in childhood obesity and is positively correlated with the rise in sucrose (table sugar) and fructose consumption, mainly present in sugar-sweetened beverages (8, 10, 26).

Despite dietary counseling, patient compliance was poor and the progressive cognitive decline culminated in OLT. Liver transplantation restores about 10% of BCKDH activity, being an effective long-term alternative to dietary treatment, sufficient to control BCAA metabolism under most circumstances, and providing protection from further cognitive decline (1, 4) and increased quality of life (1, 27, 28), which were both achieved in our patient.

However, the metabolic syndrome persisted after OLT, and significant changes in the levels of transaminases in the posttransplant period raised the hypothesis of recurrent NASH, which was confirmed by liver histology. In fact, our patient's pretransplant overweight and liver steatosis were predictive of an unfavorable outcome. It is wellestablished in the literature that pretransplant obesity, insulin resistance, hyperlipidemia, a sedentary lifestyle, and increased fat and fructose intake are among the most common risk factors for recurrent NAFLD among patients with known NASH (29–31).

Nevertheless, the early recurrence of NAFLD in the allograft was puzzling at first sight but suggested that metabolic dysregulation was capable of rapidly reproducing the disease in a healthy organ (histology of the donor's liver was reviewed and revealed no steatosis). When the diet was once again revised, the patient assumed high levels of consumption of added sugars, particularly fructose-sweetened beverages. The liver is the primary organ to metabolize fructose, and overwhelming fructose consumption is a driving force in the development of NAFLD/NASH *via de novo* lipogenesis, hepatic insulin resistance, lipotoxicity, and oxidative stress (13, 32). Fructose overconsumption also induces gut dysbiosis, visceral adiposity, and intracellular cortisol concentration (12, 33). Recent evidence suggests that the pathogenesis of NAFLD is due to the distinct characteristics of its metabolism by fructokinase C, ultimately resulting in uric acid generation that mediates fat accumulation (12, 34). Likewise, there is a strong association between high uric acid levels and pediatric NASH patients, as reported in our case.

A median fructose intake of >20 g/day or >10–30% of total energy intake induces the accumulation of fat in hepatocytes (35). Moreover, to develop a fatty liver, it usually takes at least 8–24 weeks on a high-fructose diet (9), which correlates well with the rapid development of graft steatosis in our patient. The pattern of fructose consumption is important in the pathogenesis of NAFLD because if it is accompanied by dietary fiber, fructose absorption will be slower, in contrast to diets with sugar additives and sugar-sweetened beverages, most commonly sucrose and the sweetener high-fructose corn syrup, which have the most detrimental effect (13).

Restriction of fructose consumption may ameliorate and reverse the effects on hepatic steatosis and related metabolic and biochemical parameters (12) and may be more important than weight loss for improving markers of cytolysis in children with NAFLD (36). We believe that in our patient, targeting reductions in fructose intake, particularly in beverages and processed foods, was the major therapeutic strategy to ameliorate graft steatosis. Additionally, we speculate that in this case, the consequences of excessive fructose intake and the benefits of fructose reduction may be independent of metabolic control. However, distinguishing and isolating the contribution of improved diet aspects from metabolic control may be not straightforward, particularly in the pretransplant setting. For instance, an elevated fasting blood BCAA concentration is considered a metabolic hallmark of obesity, insulin resistance, dyslipidemia, and NAFLD. Although it is not established whether BCAAs are drivers of insulin resistance and its comorbidities or only biomarkers of metabolic dysregulation (37), two recent studies reported a positive association between plasma BCAA and intrahepatic lipid content (38, 39). Therefore, it is plausible that elevated plasma BCAA levels could have been a contributing factor to insulin resistance and NAFLD in our patient. However, causality cannot be concluded since the mechanisms of elevated BCAA levels leading to hepatic fat accumulation are still unknown. Further studies with a larger cohort of MSUD subjects will be important to validate our report and evaluate the possible interaction between BCAA dyshomeostasis and nutritional status (beyond BCAA-restricted diets) in the development of NAFLD.

## Conclusion

To the best of our knowledge, this is the first case report of NAFLD in a patient with MSUD, who developed recurrent NAFLD post-liver transplantation. This pilot case report provides insights into the complexity of MSUD beyond leucine toxicity and highlights the need for the study of nutritional status and dietary intake beyond a BCAA-restricted diet.

## Data availability statement

The original contributions presented in the study are included in the article/supplementary material, further inquiries can be directed to the corresponding authors.

## Author contributions

HM-S drafted the manuscript. SF, MA, IG, MC, JRV, ES-S, and EG-M provided critical revision for important intellectual content. All authors read and approved the final version of the manuscript.

## Conflict of interest

The authors declare that the research was conducted in the absence of any commercial or financial relationships that could be construed as a potential conflict of interest. The handling editor AIL declared a past co-authorship with the authors ES and EM.

## Publisher's note

All claims expressed in this article are solely those of the authors and do not necessarily represent those of their affiliated organizations, or those of the publisher, the editors and the reviewers. Any product that may be evaluated in this article, or claim that may be made by its manufacturer, is not guaranteed or endorsed by the publisher.
